# Neutrophil Percentage-to-Albumin Ratio Predicts Contrast-Induced Acute Kidney Injury in Acute Coronary Syndrome Patients Undergoing Percutaneous Coronary Intervention

**DOI:** 10.3390/jcm14238367

**Published:** 2025-11-25

**Authors:** Hasan Can Konte, Emir Dervis, Mehmet Serkan Cetin, Omer Alyan, Dursun Aras

**Affiliations:** 1 Department of Cardiology, Istanbul Medipol University, Istanbul 34815, Türkiye; emirdervis@hotmail.com (E.D.); droalyan@yahoo.com (O.A.); drdaras@gmail.com (D.A.); 2 Department of Cardiology, Ankara Bilkent City Hospital, Ankara 06800, Türkiye; mehmetserkancetin@gmail.com

**Keywords:** acute coronary syndrome, acute kidney injury, neutrophils, serum albumin, percutaneous coronary intervention

## Abstract

**Objectives:** This study aimed to evaluate the predictive value of the neutrophil percentage-to-albumin ratio (NPAR) and other inflammatory indices for contrast-induced acute kidney injury (CI-AKI) in patients undergoing percutaneous coronary intervention (PCI) for acute coronary syndrome (ACS). **Methods:** This retrospective cohort study included 317 ACS patients (aged ≥18 years) undergoing PCI between May 2022 and July 2024 at a single center in Turkey. Patients were divided into two groups based on CI-AKI development: those who developed CI-AKI (*n* = 35, 11.1%) and those who did not (*n* = 282, 88.9%). Data on demographics, clinical variables, and laboratory parameters (complete blood count, biochemistry) were collected from medical records. Inflammatory indices (neutrophil percentage-to-albumin ratio [NPAR], neutrophil-to-lymphocyte ratio [NLR], systemic immune–inflammation index [SII], pan-immune–inflammation value [PIV], systemic inflammation response index [SIRI]) were calculated. CI-AKI was defined as a ≥0.5 mg/dL absolute or ≥25% relative increase in serum creatinine within 48–72 h after contrast exposure. **Results:** The CI-AKI group demonstrated significantly higher neutrophil counts (*p* < 0.001) and neutrophil percentages (*p* < 0.001) and lower lymphocyte counts (*p* = 0.024) compared to the non-CI-AKI group. Baseline creatinine was lower in CI-AKI patients (*p* = 0.001) but showed significantly greater post-procedural increases (*p* = 0.008). All inflammatory indices predicted CI-AKI development, with NPAR showing superior performance: NPAR (AUC = 0.896, sensitivity 82.9%, specificity 84.0%), NLR (AUC = 0.732), SII (AUC = 0.694), PIV (AUC = 0.674), and SIRI (AUC = 0.709) (all *p* < 0.001). Independent predictors of CI-AKI included NPAR >18.44 (OR = 8.511, 95% CI: 2.763–26.212, *p* < 0.001), SIRI > 2.4 × 10^3^ (OR = 2.991, *p* = 0.036), neutrophil count (OR = 1.707, *p* = 0.008), beta-blocker use (OR = 13.037, *p* = 0.016), and atrial fibrillation (OR = 8.042, *p* = 0.044). **Conclusions:** NPAR emerges as an accessible biomarker for predicting CI-AKI in ACS-PCI patients, which is also superior to other inflammation indices. We believe it is necessary to recommend its integration into risk stratification to improve outcomes among PCI recipients.

## 1. Introduction

Acute coronary syndrome (ACS), a manifestation of coronary artery disease, is a leading cause of morbidity and mortality worldwide due to its acute nature and impact on blood flow to the myocardium [1,2]. Percutaneous coronary intervention (PCI) improves outcomes by rapidly restoring coronary blood flow; however, the use of intravascular contrast agents during PCI is associated with potential adverse effects [3]. Among these, contrast-induced acute kidney injury (CI-AKI) stands out as a critical complication that negatively impacts prognosis [3,4].

Based on the definitions by the Kidney Disease: Improving Global Outcomes (KDIGO) group, CI-AKI is typically defined as an absolute increase in serum creatinine of at least 0.5 mg/dL or a relative increase of 25% from baseline within 48–72 h following contrast exposure [5]. CI-AKI is rather common and is recognized as the third most common cause of hospital-acquired AKI [3,6]. Reported incidence varies depending on patient risk profiles and procedural complexity, ranging from 5% in the general PCI population to over 20% in high-risk groups (the elderly and those with pre-existing chronic kidney disease, diabetes mellitus, or hemodynamic instability) [3,7]. CI-AKI is critical for its prognostic implications, which include major adverse cardiovascular events and mortality [2,8]. Early identification of patients at risk for CI-AKI could facilitate targeted preventive measures [3,9].

A variety of clinical scores and biochemical markers have been investigated to predict CI-AKI, including the Mehran risk score and parameters of renal function and hemodynamic status [5]. More recently, attention has shifted to the role of systemic inflammation in the pathogenesis of CI-AKI [3,6]. Consequently, simple hematological indices derived from complete blood count and routine biochemistry, such as the neutrophil-to-lymphocyte ratio (NLR), the platelet-to-lymphocyte ratio (PLR), the systemic immune–inflammation index (SII), the pan-immune–inflammation value (PIV), and the systemic inflammation response index (SIRI), have been proposed as potential predictors [10,11,12]. In this context, the neutrophil percentage-to-albumin ratio (NPAR) has recently gained interest since its numerator reflects acute inflammation while its denominator is a proxy measure of systemic illness [7]. Previous studies have associated NPAR with adverse outcomes in chronic kidney disease and cardiovascular disorders, including increased all-cause and cardiovascular mortality [7,13], but its role in predicting CI-AKI following PCI has not been clearly established [14]. The present study aimed to evaluate NPAR, alongside other established inflammatory indices, for their role in predicting the development of CI-AKI in patients undergoing PCI for ACS.

## 2. Materials and Methods

### 2.1. Study Design, Setting, and Population

This study was designed as a retrospective cohort study and was conducted at the Department of Cardiology of Istanbul Medipol University Hospital, Istanbul, Turkey. Medical records of patients admitted with ACS and treated with PCI between May 2022 and July 2024 were systematically reviewed. The study was conducted in accordance with the principles outlined in the Declaration of Helsinki. Ethical approval was obtained from the Istanbul Medipol University Non-Interventional Ethics Committee (Approval date: 31 July 2025, no: 946). Given the retrospective nature of the study, informed consent was waived for data collection from existing medical records.

The study population comprised adult patients (≥18 years) admitted with a diagnosis of ACS who underwent PCI during the study period. Inclusion criteria were (1) confirmed ACS, (2) treatment with PCI, and (3) availability of baseline and follow-up serum creatinine measurements within 48–72 h after contrast exposure. Exclusion criteria included (1) incomplete serum creatinine follow-up within 72 h, (2) patients on chronic dialysis, and (3) a history of systemic infection within the past month.

To determine the necessary sample size, power analysis was conducted based on descriptive statistics from a prior study by Li et al. [13], which reported an effect size of 0.157. Using a 95% confidence level (α = 0.05) and 80% power, the minimum required sample size was calculated as 317 patients, as determined with the PASS 2011 software (NCSS, LLC, Kaysville, UT, USA; www.ncss.com). Ultimately, 317 patients were enrolled, divided into two groups for comparison: those who developed CI-AKI post-intervention and those who did not.

### 2.2. ACS Management

The diagnosis and treatment of patients presenting with ACS were carried out in accordance with the recommendations of the current European Society of Cardiology guidelines on the management of ST-segment elevation myocardial infarction (STEMI) and non-ST-segment elevation acute coronary syndromes (NSTEMI). The STEMI definition was the presence of persistent chest pain lasting longer than 20 min accompanied by new ST-segment elevation in at least two contiguous leads or new left bundle branch block on the electrocardiogram, as well as elevated cardiac biomarkers confirming myocardial necrosis [15]. NSTEMI was defined as the presence of typical ischemic symptoms and elevated cardiac biomarkers without diagnostic ST-segment elevation on the electrocardiogram [15].

All patients underwent urgent or early PCI according to the severity and type of ACS. The amount of contrast used during PCI was recorded in milliliters (mL) and was determined by the interventional cardiologist according to lesion complexity, vessel anatomy, and procedural requirements. The duration of intervention (in minutes) was defined as the total time elapsed from arterial puncture to the removal of the guiding catheter at the end of the PCI procedure. A nonionic, low-osmolar iodinated contrast agent—iopromide (Ultravist 370; Bayer, Istanbul, Turkey)—was used for all PCI procedures.

### 2.3. Data Collection

Demographic and clinical data were collected retrospectively from all available medical records and the hospital information system. All collected data were standardized and recorded in a dedicated database for analysis. Baseline demographic information including age, sex, height, weight, and body mass index (BMI) were obtained. Clinical history comprised smoking status and comorbidities such as hypertension, diabetes mellitus, atrial fibrillation, chronic obstructive pulmonary disease, prior coronary artery disease, and previous stroke. These comorbidities were selected as they represent major cardiovascular risk factors with established associations with CI-AKI risk in the literature and were consistently recorded in our institutional database. Medication history included the use of oral antidiabetic drugs, angiotensin-converting enzyme inhibitors/angiotensin receptor blockers, diuretics, beta-blockers, calcium channel blockers, antiplatelet therapy, and statins. Echocardiographic parameters, including left ventricular ejection fraction (LVEF), were retrieved from pre-procedural assessments.

### 2.4. Laboratory Variables

Laboratory parameters were obtained from blood samples taken at baseline (pre-intervention at the emergency department) and post-intervention (at the coronary intensive care unit). Blood analyses were performed in the central laboratory of our hospital using calibrated devices. Laboratory data included complete blood count and biochemical parameters measured before and after PCI. These comprised hemoglobin, hematocrit, platelet count, mean platelet volume, platelet distribution width, white blood cell count, and differential leukocyte counts (neutrophils, lymphocytes, monocytes). Serum creatinine, estimated glomerular filtration rate (eGFR, calculated using the CKD-EPI equation), uric acid, C-reactive protein (CRP), total protein, albumin, and lipid profile (total cholesterol, high-density lipoprotein cholesterol, low-density lipoprotein cholesterol, triglycerides) were also evaluated.

### 2.5. Calculation of Inflammatory Indices

Several inflammatory indices were calculated based on the laboratory measurements:

- The NPAR was calculated by dividing neutrophil percentage (%) by serum albumin (g/dL) [14].
- The NLR was calculated as the absolute neutrophil count divided by the absolute lymphocyte count [6].
- The PLR was calculated as the platelet count divided by the lymphocyte count [6].
- The monocyte-to-lymphocyte ratio was calculated as the absolute monocyte count divided by the absolute lymphocyte count [6].
- The SII was calculated using the formula (neutrophils × platelets)/lymphocytes [11].
- The PIV was calculated as (neutrophils × platelets × monocytes) divided by lymphocytes [4].
- The SIRI was calculated as (neutrophils × monocytes) divided by lymphocytes [4].
- The CRP-to-albumin ratio (CAR) was calculated as the CRP level (mg/L) divided by serum albumin (g/dL) [5].

### 2.6. Definition of CI-AKI

Contrast-induced acute kidney injury was defined as an absolute increase in serum creatinine of ≥0.5 mg/dL or a relative increase of ≥25% from baseline within 48–72 h after contrast exposure, in accordance with established criteria [3].

### 2.7. Statistical Analysis

All analyses were conducted using IBM SPSS version 27.0 (IBM Corp., Armonk, NY, USA). *p*-values less than 0.05 were accepted as statistically significant. The distribution of continuous data was evaluated using the histograms and Q-Q plots. Descriptive statistics are presented using the mean ± standard deviation or median (25th percentile–75th percentile) based on the distribution of continuous variables. Frequency (percentage) is used for categorical variables. Between-group analysis of continuous variables was performed using Student’s *t*-test or the Mann–Whitney U test depending on the normality of the distribution. Between-group analysis of categorical variables was performed using the chi-square test or Fisher’s exact test. Repeated measurements of normally distributed variables were analyzed using the two-way repeated-measures analysis of variance (ANOVA), while those with a non-normal distribution were analyzed using the Wilcoxon signed-rank test. The performance of variables in predicting CI-AKI was evaluated using receiver operating characteristic (ROC) curve analysis. Optimal cut-off points were determined using Youden’s index. Multivariable logistic regression analysis (employing forward conditional selection) was performed to determine factors independently associated with CI-AKI.

## 3. Results

A total of 317 patients with ACS who underwent percutaneous coronary intervention were included in the study. Thirty-five patients (11.1%) were diagnosed with CI-AKI. Despite slightly higher age and greater female distribution in the CI-AKI group, the groups were similar in terms of these characteristics (*p* = 0.103 and *p* = 0.092, respectively). Atrial fibrillation (*p* = 0.043) and beta-blocker therapy (*p* < 0.001) were significantly more frequent among patients who developed CI-AKI (Table 1).

The baseline and post-procedural laboratory parameters of the study population are presented in Table 2. Hemoglobin and hematocrit levels showed a significant decline within each group after the intervention (both *p* < 0.001), but there were no significant differences between patients with and without CI-AKI in terms of changes. Patients with CI-AKI had higher absolute neutrophil counts and neutrophil percentages compared to those without CI-AKI (both *p* < 0.001). Lymphocyte counts were significantly lower in the CI-AKI group (*p* = 0.024). Regarding renal function, baseline creatinine levels were significantly lower in patients who later developed CI-AKI (*p* = 0.001). After the procedure, these patients showed a significantly greater increase in creatinine compared to the non-CI-AKI group (*p* = 0.008), with both the absolute and relative differences reaching statistical significance (*p* < 0.001). Estimated GFR values were also significantly higher in patients with CI-AKI (*p* = 0.027). Inflammatory indices including NLR, SII, PIV, SIRI, and especially NPAR were all significantly elevated in the CI-AKI group (all *p* < 0.001) (Table 2).

**Figure 1:**
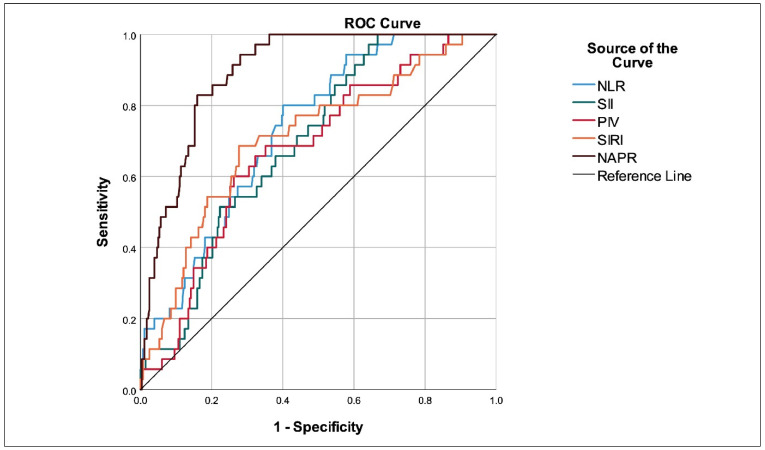
ROC curves of baseline inflammatory indices used to predict CI-AKI. All indices were calculated using pre-intervention laboratory values. NLR: neutrophil-to-lymphocyte ratio; SII: systemic immune–inflammation index; PIV: pan-immune–inflammation value; SIRI: systemic inflammation response index; NPAR: neutrophil percentage-to-albumin ratio; CI-AKI: contrast-induced acute kidney injury; ROC: receiver operating characteristic.

According to the ROC analysis, NLR (sensitivity: 80.0%; specificity: 59.9%; *p* < 0.001; AUC: 0.732), SII (sensitivity: 51.4%; specificity: 77.7%; *p* < 0.001; AUC: 0.694), PIV (sensitivity: 60.0%; specificity: 73.8%; *p* = 0.001; AUC: 0.674), SIRI (sensitivity: 68.6%; specificity: 72.3%; *p* < 0.001; AUC: 0.709), and NPAR (sensitivity: 82.9%; specificity: 84.0%; *p* < 0.001; AUC: 0.896) significantly predicted CI-AKI development after the PCI was performed for ACS (Figure 1, Table 3).

According to the multivariable logistic regression analysis results, atrial fibrillation (OR: 8.042, 95% CI: 1.057–61.184, *p* = 0.044), beta-blocker use (OR: 13.037, 95% CI: 1.611–105.510, *p* = 0.016), high neutrophil count (OR: 1.707, 95% CI: 1.152–2.530, *p* = 0.008), high SIRI (OR: 2.991, 95% CI: 1.075–8.321, *p* = 0.036), and high NPAR (OR: 8.511, 95% CI: 2.763–26.212, *p* < 0.001) were independently associated with CI-AKI. Other variables included in the analysis, such as neutrophil percentage (*p* = 0.426), lymphocyte count (*p* = 0.612), baseline creatinine (*p* = 0.152), GFR (*p* = 0.252), NLR (*p* = 0.490), SII (*p* = 0.496), and PIV (*p* = 0.937), were found to be non-significant (Table 4).

## 4. Discussion

The current study revealed that the NPAR is a robust predictor of CI-AKI development among patients with ACS who undergo PCI. Patients who developed CI-AKI also had higher levels of inflammatory indices at baseline, including neutrophil count, NLR, SII, PIV, SIRI, and particularly NPAR, compared to those without CI-AKI. Multivariable analysis showed that atrial fibrillation, beta-blocker use, elevated neutrophil counts, SIRI, and NPAR were independently associated with CI-AKI development.

CI-AKI is a common complication following PCI, with reported incidence rates ranging from 5% to 25%. It contributes to prolonged hospitalization, increased morbidity, and higher mortality [5]. Inflammatory and hematological biomarkers have been increasingly recognized for their role in predicting CI-AKI, as systemic inflammation exacerbates endothelial dysfunction and renal ischemia [16,17,18]. The NPAR, a cost-effective and readily obtainable composite index, has been shown to predict adverse outcomes in coronary artery disease, chronic heart failure, sepsis, atrial fibrillation, chronic kidney disease, and acute myocardial infarction [13,14,19,20,21,22]. In our study, patients who developed CI-AKI showed elevated NPAR levels compared to those without CI-AKI, and the predictive performance was high (sensitivity 82.9%, specificity 84.0%). These values were higher than other inflammatory indices like NLR, SII, PIV, and SIRI. Furthermore, multivariable logistic regression analysis confirmed that NPAR was strongly and independently associated with the occurrence of CI-AKI. This agrees with findings from He et al., who reported that elevated NPAR independently predicted CI-AKI and long-term mortality among patients undergoing PCI who did not have underlying kidney failure [14]. Similarly, in a prospective cohort study by Zhu et al. involving 1803 maintenance hemodialysis patients, higher NPAR tertiles were linked to increased all-cause and cardiovascular mortality, showing that NPAR remained relevant among patients with underlying renal failure [23]. A study based on NHANES data from 2009 to 2018 found that NPAR was positively associated with chronic kidney disease prevalence, independent of confounders [13]. In a larger examination of NHANES data of almost 20 years, elevated NPAR was determined to be associated with all-cause mortality with a J-shaped curve and cardiovascular disease mortality. Taken together, these data indicate that NPAR has broad utility in identifying renal injury and that it is associated with long-term prognosis [7]. As such, there have been researchers that have advocated for the use of markers like NPAR to guide preventive strategies in patients with renal injury risk [3].

Our results in this context expand the current understanding by showing that NPAR is independently associated with CI-AKI in PCI recipients. This parameter could be utilized to improve existing models like the Mehran score by adding an easily accessible biomarker. In elderly STEMI patients, Yildiz et al. reported SII and SIRI as strong predictors of CI-AKI [24]. However, it appears that younger patients may also benefit from NPAR-based risk stratification. We believe integrating NPAR into pre-PCI risk stratification could enhance early identification and preventive strategies, but these results should be supported by prospective validation in larger, multicenter trials to refine cut-off values.

We also explored the associations of various other inflammatory indices, most importantly including the NLR, SII, PIV, and SIRI, with the development of CI-AKI following PCI in ACS patients [2,25]. These indices have been proven to be useful tools in multiple diseases and settings owing to their moderate but significant relationships with adverse renal outcomes related to interventions and/or cardiovascular-associated diseases [26,27,28]. Enabling early identification of high-risk patients will allow for preventive treatment and careful intervention planning [1]. Our results revealed that all these indices were significantly elevated in the CI-AKI group compared to the non-CI-AKI group, with NLR showing an AUC of 0.732, SII an AUC of 0.694, PIV an AUC of 0.674, and SIRI an AUC of 0.709 in predicting CI-AKI; notably, multivariable logistic regression in our study identified high neutrophil count and SIRI as independent predictors, while NLR, SII, and PIV did not achieve independent significance. Ketenciler and Ada’s retrospective study of 300 patients undergoing peripheral vascular interventions reported that SII independently predicted CI-AKI with an AUC of 0.904, again supporting its link with inflammatory-driven renal injury [11]. Similarly, in a meta-analysis by Yang et al. involving 32,781 ACS patients post-PCI, NLR was associated with increased CI-AKI risk [1]. In a multicenter cohort of 30,822 CAD patients undergoing coronary angiography, Zhu et al. found that higher preoperative SII levels were linked to elevated CI-AKI risk, with a nonlinear dose–response relationship [8]. Additionally, Kurtul et al., in their analysis of 478 NSTE-ACS patients treated with PCI, reported that NLR independently predicted CI-AKI development. Interestingly, the authors described comparable AUC values to our NLR results, and we believe our results corroborate their findings [29]. These have been supported by other large-scale studies [6]. Furthermore, an overarching review of meta-analyses on this topic indicated that NLR had an AUC of approximately 0.73 in the literature to detect CI-AKI outcomes [4].

For SII, a study by Gucun et al. on 190 PCI patients yielded an independent association between high SII and CI-AKI [10]. For PIV, a retrospective analysis of NSTEMI patients undergoing PCI by Cetinkaya et al. demonstrated an independent relationship between elevated PIV and CI-AKI development, and the results were again similar to ours in terms of ROC performance [30]. In elderly STEMI patients, Yildiz et al. reported SII and SIRI as independent predictors of CI-AKI with very strong odds ratios; however, this may have been associated with the very high mean age in their study (which would be expected to reduce albumin levels, especially among those with chronic disease). The lower age group in our study is an advantage in this respect and indicates that the relationships hold true for younger patients as well [24], with support from other research [31,32] and meta-analyses [33]. A notable ACS study by Zhu et al. combined SII with CHA2DS2-VASc scores to predict CI-AKI and found that the combined approach improved accuracy [34]. Our results contribute to the literature by validating prior studies and also providing a broad comparative data set regarding the performances of different easily accessible inflammation indices in predicting CI-AKI among ACS patients undergoing PCI. Considering the strong performances of these indices (and especially NPAR), we believe it is apparent that there has been limited integration into routine use –which may negatively impact PCI outcomes as well as later morbidity.

Multivariable logistic regression analysis further identified other key independent predictors of CI-AKI, including atrial fibrillation and beta-blocker use. Identifying independent risk factors is crucial for refining CI-AKI prediction models, as it allows clinicians to account for modifiable and non-modifiable elements that influence renal vulnerability post-PCI [2,31]. Atrial fibrillation, for example, may contribute through hemodynamic instability and thromboembolic risks, while beta-blockers could exacerbate renal hypoperfusion in susceptible patients [9], and therefore, we believe combining these readily available biomarkers with other independent risk factors could greatly improve patient stratification and management—which is an approach that has been utilized rarely but with promising outcomes [34]. It is crucial to note that beta-blockers are believe to have the potential to worsen endothelial damage and inflammatory burden due to the reduced cardiac output of patients, which again provides support to the utility of inflammatory markers in assessing cardiovascular and renal-related risks [3].

While our study focused on contrast-induced acute kidney injury, we acknowledge that the etiology of AKI in patients with acute coronary syndrome undergoing PCI is likely multifactorial. Beyond contrast exposure, several other mechanisms may contribute to renal injury in this clinical setting. Patients presenting with STEMI often experience significant hemodynamic compromise, which can lead to reduced renal perfusion and ischemic injury. The acute inflammatory state characteristic of myocardial infarction may further compromise renal microcirculation, while procedural factors such as catheter manipulation and temporary reductions in systemic blood pressure during the intervention could exacerbate renal vulnerability. Hypoxia resulting from acute cardiac dysfunction represents another potential contributor to tubular damage. Despite these multiple potential mechanisms, the robust independent association we observed between inflammatory biomarkers—particularly NPAR—and AKI development underscores the central role of systemic inflammation in the pathogenesis. This suggests that inflammatory markers may serve as integrative indicators of overall physiological stress and renal risk, capturing the cumulative impact of various stressors. Our findings support the notion that targeting inflammatory pathways or identifying patients with elevated inflammatory burden could help mitigate AKI risk, regardless of the specific inciting factors.

The comparison of multiple indices in this study and the ability to compare available markers for their predictive role in CI-AKI are novel aspects of this study, as is the multifaceted analysis of risk factors. However, the retrospective design may introduce biases, such as incomplete data collection or selection bias, as patient information was gathered from records, potentially leading to inaccuracies in baseline assessments. Additionally, the relatively small number of CI-AKI cases (*n* = 35 out of 317) resulted in imbalanced group sizes, which could affect the statistical power and reliability of comparisons between the CI-AKI and non-CI-AKI groups. Confounding factors, including other potential comorbidities not systematically recorded in our database, medication adherence, or minor variations in the volume of contrast media, were not fully controlled due to the study’s retrospective nature; however, all procedures were based on institutional processes that were directly derived from international guidelines. Furthermore, as a single-center study, the generalizability of the results might be limited. It is particularly crucial to note that different procedural protocols, patient demographics, or healthcare resources may alter the results, and therefore, interpretations of the data should be made based on these factors. Finally, the current study was focused on short-term CI-AKI, and later renal complications were not examined.

## 5. Conclusions

In this retrospective study of ACS patients undergoing PCI, CI-AKI occurred in 11.1% of cases, with NPAR emerging as a superior predictor compared to other inflammatory indices like NLR, SII, PIV, and SIRI. Nonetheless, the critical influence of inflammation on AKI development was demonstrated by the fact that all inflammation indices examined appeared to be elevated in the CI-AKI group. Multivariable analysis confirmed NPAR, along with atrial fibrillation, beta-blocker use, high neutrophil count, and SIRI, as an independent risk factor for CI-AKI. NPAR is an easily accessible, cheap, and often readily available biomarker for early risk stratification in ACS-PCI settings. We strongly believe that the integration of such markers into clinical protocols could facilitate preventive strategies and directly or indirectly improve renal outcomes in this vulnerable population.

## Figures and Tables

**Table 1:** Summary of patients’ characteristics with regard to CI-AKI.

		CI-AKI		
	Total (*n* = 317)	No (*n* = 282)	Yes (*n* = 35)	*p*
Age, years	60.09 ± 12.74	59.60 ± 12.36	64.03 ± 15.09	0.103 ^†^
Sex				
Male	248 (78.23%)	225 (79.79%)	23 (65.71%)	0.092 ^§^
Female	69 (21.77%)	57 (20.21%)	12 (34.29%)	
Height, m	1.70 ± 0.07	1.70 ± 0.07	1.70 ± 0.06	0.982 ^†^
Weight, kg	78.14 ± 15.95	77.71 ± 16.20	81.60 ± 13.40	0.174 ^†^
Body mass index, kg/m^2^	27.02 ± 5.18	26.87 ± 5.25	28.23 ± 4.42	0.141 ^†^
Diagnosis				
STEMI	210 (66.25%)	188 (66.67%)	22 (62.86%)	0.795 ^§^
NSTEMI	107 (33.75%)	94 (33.33%)	13 (37.14%)	
Comorbidity				
Prior coronary artery disease	89 (28.08%)	82 (29.08%)	7 (20.00%)	0.353 ^§^
Hypertension	86 (27.13%)	73 (25.89%)	13 (37.14%)	0.226 ^§^
Diabetes mellitus	97 (30.60%)	85 (30.14%)	12 (34.29%)	0.759 ^§^
Stroke	18 (5.68%)	16 (5.67%)	2 (5.71%)	1.000 ^#^
Atrial fibrillation	13 (4.10%)	9 (3.19%)	4 (11.43%)	**0.043 ^#^**
COPD	36 (11.36%)	33 (11.70%)	3 (8.57%)	0.780 ^#^
LVEF	44.80 ± 8.50	44.76 ± 8.31	45.14 ± 9.99	0.800 ^†^
Smoking	130 (41.01%)	118 (41.84%)	12 (34.29%)	0.499 ^§^
Medication				
Oral antidiabetics	27 (8.52%)	23 (8.16%)	4 (11.43%)	0.519 ^#^
ACE inhibitors/ARB	278 (87.70%)	249 (88.30%)	29 (82.86%)	0.410 ^#^
Diuretics	61 (19.24%)	53 (18.79%)	8 (22.86%)	0.728 ^§^
Beta-blockers	179 (56.47%)	145 (51.42%)	34 (97.14%)	**<0.001 ^§^**
Calcium channel blockers	23 (7.26%)	20 (7.09%)	3 (8.57%)	0.729 ^#^
Antiplatelets	228 (71.92%)	204 (72.34%)	24 (68.57%)	0.788 ^§^
Statins	112 (35.33%)	99 (35.11%)	13 (37.14%)	0.960 ^§^
Contrast amount, mL	120 (100–120)	120 (100–120)	120 (115–120)	0.590 ^‡^
Duration of intervention, min	59.44 ± 15.29	59.17 ± 15.05	61.63 ± 17.17	0.370 ^†^

Descriptive statistics are presented using mean ± standard deviation for normally distributed continuous variables, median (25th percentile–75th percentile) for non-normally distributed continuous variables, and frequency (percentage) for categorical variables. ^†^ Student’s *t*-test; ^‡^ Mann–Whitney U test; ^§^ chi-square test; ^#^ Fisher’s exact test. Statistically significant *p* values are shown in bold. Abbreviations: ACEi/ARB: angiotensin-converting enzyme inhibitors/angiotensin receptor blockers; AF: atrial fibrillation; BMI: body mass index; CI-AKI: contrast-induced acute kidney injury; COPD: chronic obstructive pulmonary disease; LVEF: left ventricular ejection fraction; NSTEMI: non-ST-segment elevation myocardial infarction; PCI: percutaneous coronary intervention; STEMI: ST-segment elevation myocardial infarction.

**Table 2:** Summary of laboratory measurements with regard to CI-AKI.

		CI-AKI		
	Total (*n* = 317)	No (*n* = 282)	Yes (*n* = 35)	*p*
Hemoglobin, g/dL				
Baseline	14.09 ± 1.89	14.13 ± 1.90	13.81 ± 1.81	0.341 ^§^
Post-intervention	13.17 ± 1.88	13.20 ± 1.89	12.93 ± 1.84	0.415 ^§^
*p* (within groups)	**<0.001 ^§^**	**<0.001 ^§^**	**<0.001 ^§^**	
Difference ^(1)^	−0.92 ± 0.89	−0.92 ± 0.88	−0.88 ± 1.03	0.766 ^§^
Hematocrit, %				
Baseline	42.8 (39.7–45.6)	42.75 (39.9–45.7)	43.3 (36.7–44.9)	0.268 ^‡^
Post-intervention	39.6 (35.7–43.2)	39.7 (35.8–43.2)	38.5 (34.9–41.8)	0.343 ^‡^
*p* (within groups)	**<0.001 ^#^**	**<0.001 ^#^**	**<0.001 ^#^**	
Difference ^(1)^	−2.6 (−5.2–0.0)	−2.6 (−5.5–0.0)	−2.1 (−4.1–0.6)	0.487 ^‡^
Platelet, 10^3^/μL	261.69 ± 63.68	263.54 ± 63.74	246.80 ± 62.10	0.143 ^†^
MPV, fL	10.11 ± 1.69	10.10 ± 1.76	10.25 ± 0.93	0.435 ^†^
PDW, fL	11.4 (10.2–12.6)	11.4 (10.1–12.6)	11.4 (10.5–12.1)	0.848 ^‡^
WBC, 10^3^/μL	10.01 (8.6–11.43)	10.07 (8.55–11.47)	9.89 (8.72–11.15)	0.539 ^‡^
Neutrophil, 10^3^/μL	5.82 (5.48–6.21)	5.75 (5.42–6.07)	7.97 (7.52–8.72)	**<0.001 ^‡^**
Neutrophil, %	62.09 ± 13.70	59.73 ± 12.21	81.13 ± 9.65	**<0.001 ^†^**
Lymphocyte, 10^3^/μL	2.53 ± 1.13	2.57 ± 1.15	2.18 ± 0.91	**0.024 ^†^**
Monocyte, 10^3^/μL	0.70 (0.54–0.89)	0.71 (0.55–0.90)	0.68 (0.48–0.87)	0.537 ^‡^
Creatinine, mg/dL				
Baseline	0.93 (0.78–1.05)	0.95 (0.80–1.06)	0.78 (0.67–0.93)	**0.001 ^‡^**
Post-intervention	0.97 (0.84–1.13)	0.97 (0.83–1.12)	1.04 (0.89–1.36)	**0.008 ^‡^**
*p* (within groups)	**<0.001 ^#^**	**<0.001 ^#^**	**<0.001 ^#^**	
Difference ^(1)^	0.07 (0.02–0.11)	0.06 (0.01–0.09)	0.26 (0.20–0.43)	**<0.001 ^‡^**
Difference, % ^(1)^	0.07 (0.02–0.12)	0.07 (0.01–0.09)	0.32 (0.28–0.39)	**<0.001 ^‡^**
GFR, mL/min/1.73 m^2^	83 (72–104)	82.5 (72–103)	98 (80–115)	**0.027 ^‡^**
Uric acid, mg/dL	5.5 (4.4–7.3)	5.6 (4.4–7.8)	5.3 (4.3–6.1)	0.135 ^‡^
CRP, mg/L	6.55 (2.85–17.20)	6.68 (2.85–17.55)	4.46 (2.29–12.30)	0.187 ^‡^
Total protein, g/dL	6.56 (6.10–6.96)	6.56 (6.09–6.97)	6.42 (6.10–6.72)	0.283 ^‡^
Albumin, g/dL	3.98 ± 0.36	3.99 ± 0.36	3.93 ± 0.35	0.422 ^†^
Total bilirubin, mg/dL	0.47 (0.35–0.70)	0.47 (0.35–0.70)	0.57 (0.32–0.78)	0.329 ^‡^
Direct bilirubin, mg/dL	0.17 (0.12–0.24)	0.17 (0.12–0.24)	0.16 (0.12–0.25)	0.392 ^‡^
Total cholesterol, mg/dL	179.53 ± 37.07	179.92 ± 37.11	176.40 ± 37.09	0.597 ^†^
HDL, mg/dL	37 (32–44)	37 (31–44)	39 (33–46)	0.177 ^‡^
LDL, mg/dL	114.60 ± 31.25	115.12 ± 31.27	110.43 ± 31.24	0.403 ^†^
Triglyceride, mg/dL	119 (80–180)	121.5 (82–179)	110 (64–202)	0.487 ^‡^
Fasting glucose, mg/dL	114 (93–151)	114 (93–151)	119 (95–179)	0.321 ^‡^
NLR	2.54 (1.72–3.70)	2.44 (1.63–3.48)	3.55 (2.86–5.18)	**<0.001 ^‡^**
PLR	108.79 (75.91–162.34)	105.96 (75.41–162.57)	124.19 (88.58–152.13)	0.367 ^‡^
MLR	0.29 (0.20–0.43)	0.28 (0.20–0.43)	0.37 (0.21–0.45)	0.155 ^‡^
SII, ×10^3^	674.72 (451.82–954.96)	636.81 (432.71–910.99)	942.63 (624.92–1162.24)	**<0.001 ^‡^**
PIV, ×10^6^	471.13 (299.13–685.95)	444.31 (293.58–644.52)	656.75 (423.63–878.31)	**<0.001 ^‡^**
SIRI, ×10^3^	1.75 (1.24–2.68)	1.67 (1.22–2.60)	2.82 (1.80–3.55)	**<0.001 ^‡^**
NPAR	15.73 ± 3.78	15.10 ± 3.40	20.75 ± 2.90	**<0.001 ^†^**
CAR	1.64 (0.70–4.45)	1.69 (0.70–4.55)	1.16 (0.53–3.84)	0.223 ^‡^

Descriptive statistics are presented using mean ± standard deviation for normally distributed continuous variables and median (25th percentile–75th percentile) for non-normally distributed continuous variables. ^†^ Student’s *t*-test; ^‡^ Mann–Whitney U test; ^§^ two-way repeated-measures analysis of variance (ANOVA); ^#^ Wilcoxon signed-rank test. Statistically significant *p* values are shown in bold. ^(1)^ Difference between post-intervention and baseline; negative values represent decreases and positive values represent increases. Laboratory parameters other than hemoglobin, hematocrit, and creatinine represent baseline (pre-intervention) values obtained at hospital admission. Abbreviations: CAR: C-reactive protein-to-albumin ratio; CI-AKI: contrast-induced acute kidney injury; CRP: C-reactive protein; GFR: glomerular filtration rate; HDL: high-density lipoprotein; LDL: low-density lipoprotein; MLR: monocyte-to-lymphocyte ratio; MPV: mean platelet volume; NPAR: neutrophil percentage-to-albumin ratio; NLR: neutrophil-to-lymphocyte ratio; PDW: platelet distribution width; PIV: pan-immune–inflammation value; PLR: platelet-to-lymphocyte ratio; SII: systemic immune–inflammation index; SIRI: systemic inflammation response index; WBC: white blood cell.

**Table 3:** Performance of baseline inflammatory indices in predicting CI-AKI, ROC curve analysis.

	Cut-Off	Sensitivity	Specificity	Accuracy	PPV	NPV	AUC (95% CI)	*p*
NLR	>2.75	80.00%	59.93%	62.15%	19.86%	96.02%	0.732 (0.657–0.807)	**<0.001**
SII (×10^3^)	>940	51.43%	77.66%	74.76%	22.22%	92.80%	0.694 (0.618–0.770)	**<0.001**
PIV (×10^6^)	>621.7	60.00%	73.76%	72.24%	22.11%	93.69%	0.674 (0.586–0.762)	**0.001**
SIRI (×10^3^)	>2.4	68.57%	72.34%	71.92%	23.53%	94.88%	0.709 (0.616–0.802)	**<0.001**
NPAR	>18.44	82.86%	84.04%	83.91%	39.19%	97.53%	0.896 (0.856–0.936)	**<0.001**

Statistically significant *p* values are shown in bold. All inflammatory indices were calculated using baseline (pre-intervention) laboratory values obtained at hospital admission. Abbreviations: NLR: neutrophil-to-lymphocyte ratio; SII: systemic immune–inflammation index; PIV: pan-immune–inflammation value; SIRI: systemic inflammation response index; NPAR: neutrophil percentage-to-albumin ratio; AUC: area under ROC curve; CI: confidence interval; NPV: negative predictive value; PPV: positive predictive value; ROC: receiver operating characteristic.

**Table 4:** Significant factors independently associated with CI-AKI in the multivariable logistic regression analysis.

	β Coefficient	Standard Error	*p*	Exp(β)	95% CI for Exp(β)	
Atrial fibrillation, Yes	2.085	1.035	**0.044**	8.042	1.057	61.184
Beta-blockers use, Yes	2.568	1.067	**0.016**	13.037	1.611	105.510
Neutrophil, 10^3^	0.535	0.201	**0.008**	1.707	1.152	2.530
SIRI, >2.4 × 10^3^	1.096	0.522	**0.036**	2.991	1.075	8.321
NPAR, >18.44	2.141	0.574	**<0.001**	8.511	2.763	26.212
Constant	−9.385	1.514	<0.001			

Statistically significant *p* values are shown in bold. Abbreviations: CI: confidence interval; NPAR: neutrophil percentage-to-albumin ratio; Nagelkerke R^2^: Nagelkerke coefficient of determination; SIRI: systemic inflammation response index.

## Data Availability

The data that support the findings of this study are available from the corresponding author upon reasonable request.

## Abbreviations

NPAR	neutrophil percentage-to-albumin ratio
CI-AKI	contrast-induced acute kidney injury
PCI	percutaneous coronary intervention
ACS	acute coronary syndrome
NLR	neutrophil-to-lymphocyte ratio
SII	systemic immune–inflammation index
PIV	pan-immune–inflammation value
SIRI	systemic inflammation response index
AUC	area under curve
KDIGO	Kidney Disease: Improving Global Outcomes
STEMI	ST-segment elevation myocardial infarction
NSTEMI	non-ST-segment elevation acute coronary syndromes
BMI	body mass index
LVEF	left ventricular ejection fraction
eGFR	estimated glomerular filtration rate
CRP	C-reactive protein
CAR	CRP-to-albumin ratio
ANOVA	analyzed using the two-way repeated measures analysis of variance
ROC	receiver operating characteristic
ACEi/ARB	angiotensin-converting enzyme inhibitors/angiotensin receptor blockers
AF	atrial fibrillation
COPD	chronic obstructive pulmonary disease
GFR	glomerular filtration rate
HDL	high-density lipoprotein
LDL	low-density lipoprotein
MLR	monocyte-to-lymphocyte ratio
MPV	mean platelet volume,
PDW	platelet distribution width
WBC	white blood cell
